# Antinociception induced by chronic glucocorticoid treatment is correlated to local modulation of spinal neurotransmitter content

**DOI:** 10.1186/1744-8069-5-41

**Published:** 2009-07-24

**Authors:** Filipa Pinto-Ribeiro, Vitor Moreira, José M Pêgo, Pedro Leão, Armando Almeida, Nuno Sousa

**Affiliations:** 1 Life and Health Science Research Institute, School of Health Sciences, University of Minho, Campus de Gualtar, 4710-057 Braga, Portugal

## Abstract

**Background:**

While acute effects of stress on pain are well described, those produced by chronic stress are still a matter of dispute. Previously we demonstrated that chronic unpredictable stress results in antinociception in the tail-flick test, an effect that is mediated by increased levels of corticosteroids. In the present study, we evaluated nociception in rats after chronic treatment with corticosterone (CORT) and dexamethasone (DEX) in order to discriminate the role of each type of corticosteroid receptors in antinociception.

**Results:**

Both experimental groups exhibited a pronounced antinociceptive effect after three weeks of treatment when compared to controls (CONT); however, at four weeks the pain threshold in CORT-treated animals returned to basal levels whereas in DEX-treated rats antinociception was maintained. In order to assess if these differences are associated with altered expression of neuropeptides involved in nociceptive transmission we evaluated the density of substance P (SP), calcitonin gene-related peptide (CGRP), somatostatin (SS) and _B2_-γ-aminobutiric acid receptors (GABA_B2_) expression in the spinal dorsal horn using light density measurements and stereological techniques. After three weeks of treatment the expression of CGRP in the superficial dorsal horn was significantly decreased in both CORT and DEX groups, while GABA_B2 _was significantly increased; the levels of SP for both experimental groups remained unchanged at this point. At 4 weeks, CGRP and SP are reduced in DEX-treated animals and GABA_B2 _unchanged, but all changes were restored to CONT levels in CORT-treated animals. The expression of SS remained unaltered throughout the experimental period.

**Conclusion:**

These data indicate that corticosteroids modulate nociception since chronic corticosteroid treatment alters the expression of neuropeptides involved in nociceptive transmission at the spinal cord level. As previously observed in some supraspinal areas, the exclusive GR activation resulted in more profound and sustained behavioural and neurochemical changes, than the one observed with a mixed ligand of corticosteroid receptors. These results might be of relevance for the pharmacological management of certain types of chronic pain, in which corticosteroids are used as adjuvant analgesics.

## Background

Nociception can be modulated at different levels of the CNS through facilitating (pronociceptive) or inhibiting (antinociceptive) central actions [1-3]. One of the levels where nociceptive modulation takes place is in laminae I–II of the spinal dorsal horn [4], where nociceptors synapse upon interneurons and projection neurons [5,6]. The transmission of nociceptive information in the dorsal horn involves several events, neuropeptides and fibres. After peripheral noxious stimulation of unmyelinated nociceptors the release of calcitonin gene-related peptide (CGRP) [7], substance P (SP) [8] and somatostatin (SS) [4,9] is increased although it remains largely unchanged after innocuous stimulation or stimulation of large myelinated fibres [8,9]. Spinal nociceptive neurons that are excited by CGRP and SP [10,11] receive numerous synaptic contacts from primary afferent terminals colocalizing these neurotransmitters, whereas non-nociceptive neurons lack synaptic input from boutons with both peptides [12]. Spinal SS [13] and GABA [14] have an inhibitory effect on nociceptive neurons, being present mainly in fibres belonging to local inhibitory interneurons [15].

Acute stress induces analgesia but the effects of chronic stress in nociception are still controversial, with studies reporting hyperalgesia after prolonged stress [16], while others observed analgesia [17]. Recently, we demonstrated that animals submitted to chronic unpredictable stress display antinociception in the tail-flick test [18]; since the plasmatic levels of corticosteroids were increased throughout the entire experimental period, we implicated these hormones in that phenomenon. Corticosteroids can bind to two types of corticosteroid receptors, mineralocorticoid (MR) and glucocorticoid (GR) receptors. In basal conditions, MR display greater occupancy than GR; thus, conditions resulting in elevation of corticosteroids, e.g. stress, will result mainly in increased activation of GR. Importantly, the spinal cord is a corticoid-responsive tissue [19] and within the spinal cord the greatest density of GR and MR occurs in laminae I–II [20]. Of notice, CGRP and SP (but not SS) coexist with corticosteroid receptors in neurons of dorsal root ganglia [21] and some studies demonstrate that an imbalanced corticosteroid milieu may affect neuropeptide content in the DRG [22,23]. Importantly, corticosteroids are often used as adjuvant analgesics in the management of several types of pain [24-26]. Taken together, these findings predict a potential influence of corticosteroids in the modulation of spinal nociceptive transmission.

In the premise that a distinctive activation of MR or GR could be responsible for altered levels of neuropeptides involved in spinal nociceptive transmission and, consequently, for diverse pain-like effects we evaluated the density of CGRP, SP, SS and GABA_B2 _innervation in the spinal dorsal horn of animals submitted to prolonged administration of CORT (activating both MR and GR) and DEX (a selective ligand of GR). These data were correlated with pain-like behaviour measured through the tail-flick and hot-plate tests.

## Results

### Pain-like Behaviour

#### Evolution within groups during the experimental period

Analysis of TF and HP latency in CONT revealed no significant differences between testing sessions throughout the experimental period (ANOVA_rm_, TF, P = 0.29 and HP, P = 0.60).

#### Tail-flick test

**Figure 1:**
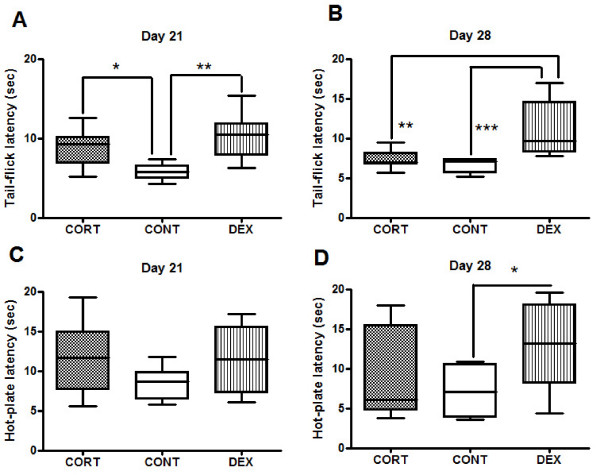
**Nociceptive behaviour**. Tail (A, B) and paw (C, D) withdrawal latency after chronic corticosteroid treatment for 21 (1) and 28 (2) days with CORT and DEX. Both CORT and DEX groups display higher TF latencies after 21 days of treatment (A, B) although this effect is only sustained by DEX group at the end of the experiment (B); note that only DEX induces an increase in hind-paw latency and only after 28 days of treatment (D). (*p < 0.05, **p < 0.01 and ***p < 0.001).

The chronic subcutaneous administration of CORT and DEX resulted in a significant decrease in pain-like behaviour. Statistical data indicate that both CORT and DEX induced a significant increase in TF latencies on day 21 (ANOVA_ow_, P = 0.002, *pos-hoc *Bonferroni, CORT × CONT, p < 0.05; DEX × CONT, p < 0.01) (Fig. 1A). However, with the prolongation of the treatment (day 28) only subjects under DEX treatment maintained the significant increase in TF thresholds; in contrast in CORT-treated animals nociceptive behaviour decreased slightly (ANOVA_ow_, P = 0.0003, *pos-hoc *Bonferroni, CORT × CONT, p > 0.05, DEX × CONT, p < 0.001 and DEX × CORT, p < 0.001) (Fig. 1B).

#### Hot-plate test

The prolonged administration of DEX but not CORT resulted in a significant decrease in nociceptive behaviour. Statistical data indicates that HP latencies are significantly increased in DEX-treated animals on day 28 (ANOVA_ow_, P = 0.02, *pos-hoc *Bonferroni, DEX × CONT, p < 0.05) (Fig. 1D). Contrary to what was observed for the TF test, no differences between groups were observed on day 21, although a trend towards an antinociceptive effect was already observed (ANOVA_ow_, P = 0.07) (Fig. 1C).

### Neurotransmitter Spinal Innervation

All statistical data presented in this section referring to immunoreactivity evaluation is based on the study of the lumbar portion of the spinal cord as no differences in neurotransmitter-IR were found between cervical and lumbar portions.

#### Stereology

**Figure 2:**
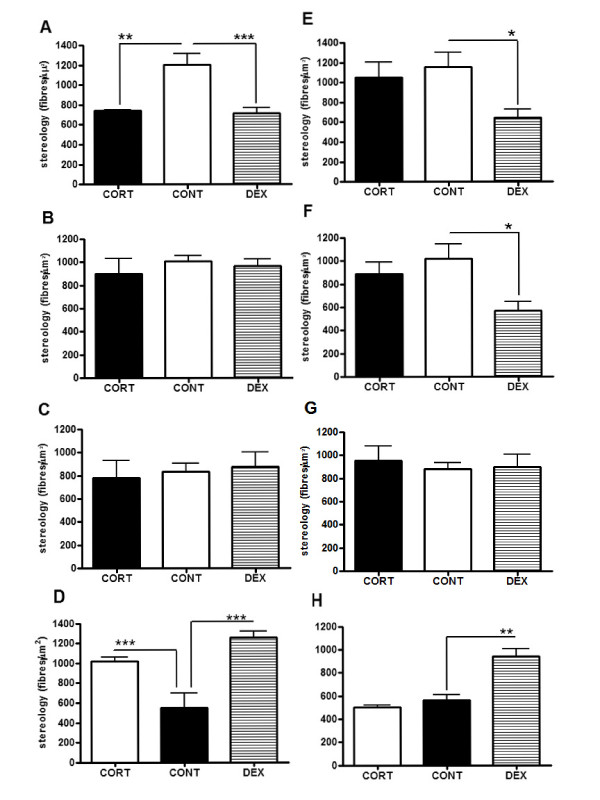
**Neuropeptide and receptor expression in the spinal dorsal horn**. Immunoreactive content in the dorsal horn of the spinal cord after 21 and 28 days of chronic corticosteroid treatment. (CGRP(A, E), SP(B, F), SS(C, G) and GABA_B2_(D, H); *p < 0.05, **p < 0.01 and ***p < 0.001).

The stereological analysis of CGRP-, SP-, SS- and GABAB_2_-IR in the spinal dorsal horn after prolonged CORT and DEX treatment is summarized in figure 2. The expression of CGRP-IR was significantly decreased in both DEX and CORT-treated animals when compared to CONT on day 21 (ANOVA_2 w_, p < 0.0001, pos-hoc Bonferroni, CORT × CONT, p < 0.01 and DEX × CONT, p < 0.001) (Fig. 2A) although this effect was sustained only in DEX animals on day 28 (ANOVA_2 w_, p < 0.036, pos-hoc Bonferroni, DEX × CONT, p < 0.05) (Fig. 2E). The level of SP-IR in CORT and DEX was not significantly different from CONT on day 21 (ANOVA_2 w_, P = 0.70) (Fig. 2B) but in DEX-treated animals there was a significant decrease in SP expression on day 28 (ANOVA_2 w_, P = 0.033, pos-hoc Bonferroni, DEX × CONT, p < 0.05) (Fig. 2F). No changes were observed between experimental groups in what concerns SS-IR in the spinal dorsal horn (ANOVA_2 w_, day 21, P = 0.86 and day 28, P = 0.88) (Figs. 2C, G). In DEX-treated animals GABA_B2_-IR is significantly increased in both 21 and 28 days (ANOVA_2 w_, p < 0.0001, DEX × CONT, day 21, p < 0.001 and day 28, p < 0.01). A similar increase was observed in the CORT-group on day 21 (ANOVA_2 w_, p < 0.0001, CORT × CONT, day 21, p < 0.001 and day 28, p > 0.05) but GABA_B2 _to returned to basal levels on day 28 (Figs. 2D, H).

#### Densitometric Measurements

**Figure 3:**
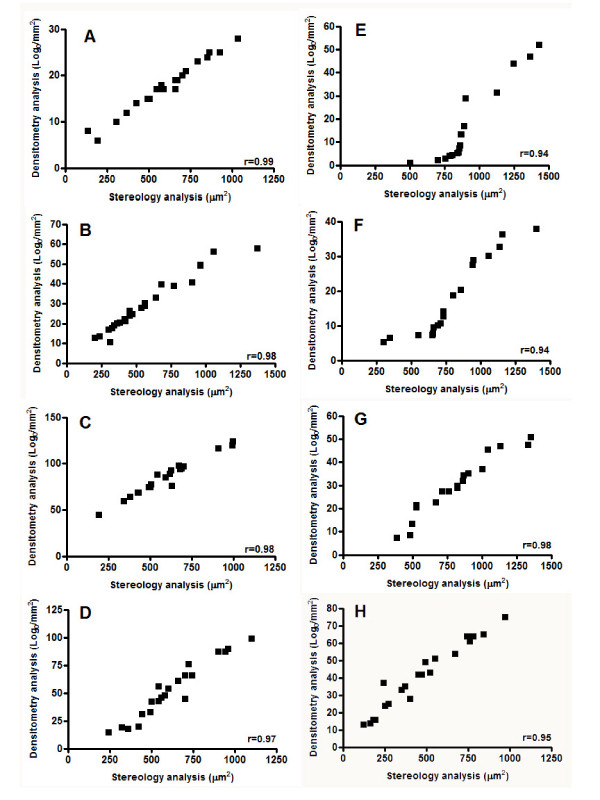
**Pearson correlation between densitometry and stereology quantification methods**. Pearson correlation for validation of densitometric versus stereologic quantification methods on days 21(A-D) and 28(E-H). (SS(A, E), SP(B, F), CGRP(C, G) and GABA_B2_(D, H)).

The results obtained through the densitometric analysis of CGRP, SP, SS and GABAB_2_-IR were closely related to those obtained through the stereological quantification both for day 21 (Pearson analysis, CGRP_21 days_, r = 0.98 and p < 0.0001; SP_21 days_, r = 0.98 and p < 0.0001; SS_21 days_, r = 0.99 and p < 0.0001; GABA_B2,21 days_, r = 0.97 and p < 0.0001) (Figs. 3A, B, C, D) and day 28 (Pearson analysis, CGRP_28 days_, r = 0.94 and p < 0.0001; SP_28 days_, r = 0.98 and p < 0.0001; SS_28 days_, r = 0.94 and p < 0.0001; GABA_B2,28 days_, r = 0.95 and p < 0.0001) (Figs. 3E, F, G, H).

## Discussion

The present study demonstrates that prolonged administration of corticosteroids decreases nociception. The antinociceptive effect reflects both a decrease of pronociceptive neuropeptide expression and an increased availability of GABA receptors in laminae I–II of the spinal dorsal horn. After 21 days of treatment the decrease in pain-like behaviour was correlated with a decrease in CGRP and an increase in GABA_B2 _receptors in the spinal cord of CORT and DEX treated-animals. Interestingly, the antinociceptive effect in the CORT-group vanished after four weeks of treatment (which was paralleled by a restoration of CGRP and GABA_B2 _expression towards control levels) while it remained unchanged in DEX-treated rats (which were correlated with a decrease in spinal content of both CGRP and SP and increased availability of GABA_B2_). These findings confirm that corticosteroid receptors play a crucial role in the mediation of pain transmission at the spinal cord level.

Pain perception involves the transmission of nociceptive messages from the periphery to the CNS. This transmission can be modulated by acute [16] and chronic stress [16,17]. Recently, we showed that chronic unpredictable stress, which results in a prolonged elevation of plasmatic glucocorticoid (GC) levels, decreases pain-like behaviour [18]. Most actions mediated by chronic stress are attributed to hypercortisolemia, as the increased secretion of corticosteroids characterizes the sustained phase of the stress response [27]. Because corticosteroids can bind to two types of receptors we decided to further explore the role of each of these corticosteroid receptors on the nociceptive modulation. It is important to note at this point, that the confounding effect of drug potency has been considered, as the doses of each corticosteroid were adjusted accordingly to their glucocorticoid potency. Thus, in this experimental paradigm CORT treatment differs from DEX administration basically in terms of MR activation: while CORT treatment activates these receptors, DEX does not bind to MR and because it shuts-off the endogenous secretion of corticosteroids, MR remains unoccupied [28].

The results observed after prolonged daily treatment with corticosteroids demonstrate that these steroids promote antinociception. GR are likely to mediate this phenomenon since a similar response was observed in DEX-treated animals. The TF test evaluates a spinally organized reflex [29,30] mediated by C-fibres innervating the tail [31,32] and motoneurons innervating the three sets of back muscles that control tail movements [33-35]. In contrast, the HP test involves a supraspinally integrated response, and thus, represents a more complex behavioural response [36]. Such difference in the neuroanatomical substrates implicated in both tests might explain why there was only a trend towards increased HP latencies after 21 days of CORT and DEX treatments.

It is admissible that the influence of GC upon neuropeptidergic innervation results both from direct and indirect actions. Indirect actions may result from altered availability of GABA_B2 _receptors in CORT and DEX treated animals on day 21, as GABA_B _receptors are well known players in pain modulation [15,37]. Moreover Kangrga and collegues [38] described that the antinociceptive effect of GABAergic transmission in the spinal dorsal horn results from presynaptic inhibition of the release of excitatory amino acids and neurotransmitters from the primary afferents [14,39] which is in accordance with our observations that CGRP, a pronociceptive neuropeptide, is decreased in both CORT- and DEX-treated groups. An alternative indirect action of glucocorticoids might occur through the modulatory actions of arachidonic pathways which down-regulate nerve growth factor (NGF); this, in turn, is known to exert an inhibitory effect in both the accumulation and release of CGRP mRNA in nociceptors [40,41]. In parallel, the direct modulation of glucocorticoids can be ascribed to the fact that approximately one third of the afferents that are immunoreactive to SP or CGRP, also display immunoreactivity to GR [21]. Thus, it is plausible to assume that GR activation of nuclear responsive elements alters the expression of such transmitters in spinal dorsal horn afferents. This hypothesis is further supported by the fact that it was recently shown that stressors decrease CGRP expression in the frontal cortex, hippocampus, occipital cortex and hypothalamus [42].

Curiously, the dissimilarity in pain-like behaviour observed between CORT and DEX groups after 28 days of treatment, suggests that other mechanisms involving MR activation are implicated in the modulation of pain. In fact, the behavioural differences observed between CORT and DEX treatment at 28 days were paralleled by distinct patterns in CGRP, SP and GABA_B2 _expression in the superficial dorsal horn: while in CORT-treated animals the expression of both CGRP and GABA_B2 _was restored to control levels, DEX treatment resulted in a decreased expression of SP and CGRP and sustained increase in GABA_B2_. The explanations for such discrepancy are more complex, as besides the local effects at the spinal cord level, they might involve alterations at the supraspinal level. Indeed, there is a complex feedback system between the neurotransmitters herein studied and GC involving supraspinal processing that is regulated by MR. There is evidence that the activation of MR is correlated with GABA modulation [43] in lamina II [44] of the spinal cord, namely in interneurones [45], and in other supraspinal pain modulating areas such as the rostroventral lateral medulla (RVM) [46] or the periaductal grey matter (PAG) [47]. This effect of GABAergic transmission appears to selectively inhibit the release of SP, but not of CGRP, [15] which may account at least partly for the differences observed between the groups. Another alternative, but not exclusive, mechanism to explain the differential effect of DEX and CORT upon neuropeptidergic spinal expression derives from the specific modulatory effects of MR upon preprotachykinin (PPT), the precursor of SP expression; in fact, MR activation has been shown to positively regulate (up to 50%) mRNA PPT expression in the nervous tissues [48]. The more persistent changes in pain perception induced by DEX treatment and measured by an increase in both the TF and HP latency at day 28 might therefore result from a decrease in SP fibre innervation in the spinal dorsal horn.

Contrary to CGRP, SP and GABA_B2_, no effect of GC was observed on the spinal levels of SS. This differential change observed between these neuropeptides illustrates the selectivity of this process, and is likely to be related with the lack of coexistence of corticosteroid and SS in the spinal cord [21]. Interestingly, different neurotransmitters are associated with different roles in pain modulation [5,49]. In contrast to CGRP/SP, SS is a tonic inhibitor of peripheral nociceptors [50]. Thus, the data herein reported suggests that the effects of chronic corticosteroid treatment on pain perception are associated with changes in the nociceptive transmitting system (CGRP/SP) but would not involve specific alterations in the spinal intrinsic modulatory system (SS).

In addition to their presence in the spinal dorsal horn, both glucocorticoid- [51] and mineralocorticoid- [52] receptors are present also in neurons of a large number of supraspinal sites along the rostrocaudal extent of the neuraxis in the rat. These include several forebrain and brainstem components of the supraspinal pain control system, including areas like the anterior cingulate cortex [53], amygdala [54], paraventricular hypothalamic nucleus [55], periaqueductal grey matter [56], locus coeruleus [57], rostral ventromedial medulla [58], dorsal reticular nucleus [59] and caudal ventrolateral medulla [60]. Taking into account data obtained in the present study on the effect of corticosteroid manipulation upon spinal neurotransmitter content, future studies should explore alterations induced at supraspinal levels. Accordingly, profound structural, physiological and neurochemical alterations have been observed at different forebrain areas following chronic manipulation of corticosteroids [30,61-63].

## Conclusion

The present study shows that corticosteroids modulate nociception by altering the expression of neuropeptides involved in nociceptive transmission at the spinal cord level. Moreover, we demonstrate differential modulatory actions of different ligands of corticosteroid receptors, which are of relevance for the pharmacological management of those conditions involving chronic pain, in which corticosteroids are recommended as adjuvant analgesics.

## Methods

### Subjects

Wistar Han rats obtained from Charles Rivers (Barcelona, UE), weighting between 200–240 g, at the beginning of the experiment, were housed in groups of three in standard polycarbonate cages (45.4 × 25.5 × 20 cm). The light cycle was 12:12 h with lights on at 9:00 am and housing was maintained at 22°C and 30% relative humidity. Water and food were available ad libitum. All regulations determined by the local veterinarian committee (in accordance to the European Community Council Directive 86/609/EEC) concerning the handling of laboratory animals and the international ethical guidelines for the study of experimental pain in conscious animals were followed [64].

### Chronic corticosteroid treatment

Corticosterone, dexamethasone and sesame oil were acquired from Sigma (St Louis, MO, USA). Subjects were assigned to one of the following three groups (n = 24):

(i) Controls (CONT). Rats were submitted to vehicle injection (0.5 ml sesame oil) everyday (05:00 pm), during 3 weeks (n = 4) and 4 weeks (n = 4).

(ii) Corticosterone-treated (CORT). Rats were submitted over a period of 3 weeks (n = 4) and 4 weeks (n = 4) to a daily subcutaneous injection (05:00 pm) of 40 mg/kg dose of 4-Pregnene-11β,21 diol-3,20-dione in sesame oil.

(iii) Dexamethasone-treated (DEX). Rats were submitted over a period of 3 weeks (n = 4) and 4 weeks (n = 4) to a daily subcutaneous injection (05:00 pm) of 300 μmg/kg dose of 9α-fluoro-16α-methylpredenisolone in sesame oil.

### Nociceptive testing

Pain-like behaviour was analyzed for each animal using the tail-flick (TF) and the hot-plate (HP) tests. In the TF (Ugo Basile, Comerio, Italy) the time spent the start of the stimulus and the withdrawal of the tail (nociceptive latency) was recorded, whereas in the HP (Ugo Basile, Comerio, Italy) as the heating plate was kept at a constant temperature of 54 ± 0.5°C, it was the latency for hind paw licking or jumping was recorded.

**Figure 4:**
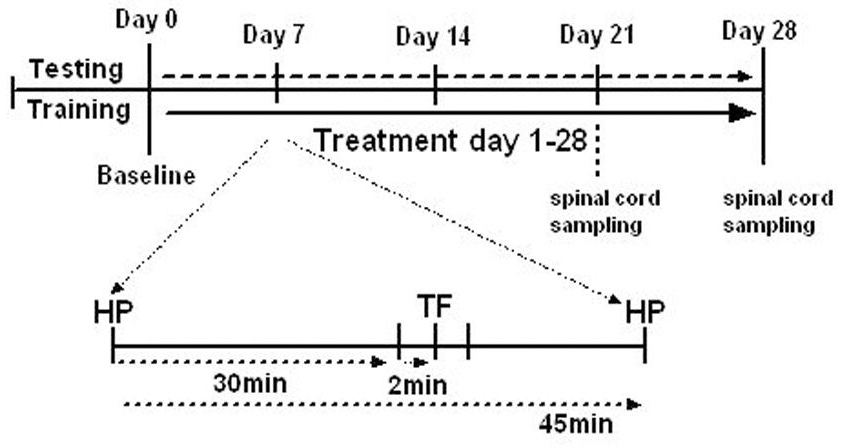
**Time course of testing and sampling sessions throughout the four week experimental period**. Within a testing session, tail-flick and hot-plate tests were performed according to the time course example for day 7.

In order to determine the nociceptive threshold, rats were tested before corticosteroid administration (day 0) and on days 7, 14, 21 and 28 of the treatment; each testing day animals were submitted (11:00 am) to 3 TF tests, within a 2 min interval, and 2 HP test, with 45 min interval (Fig. 4). To avoid bias related with the handling and testing of the rats, a one-week period prior to the first nociceptive test was established for the habituation of the animals to the behavioural test equipment and the researcher. Animals were placed daily in the test room for 2 h followed by a 10 minute handling and 1 minute training session in the TF and the HP apparatus (without performing the test).

### Immunocytochemistry

**Figure 5:**
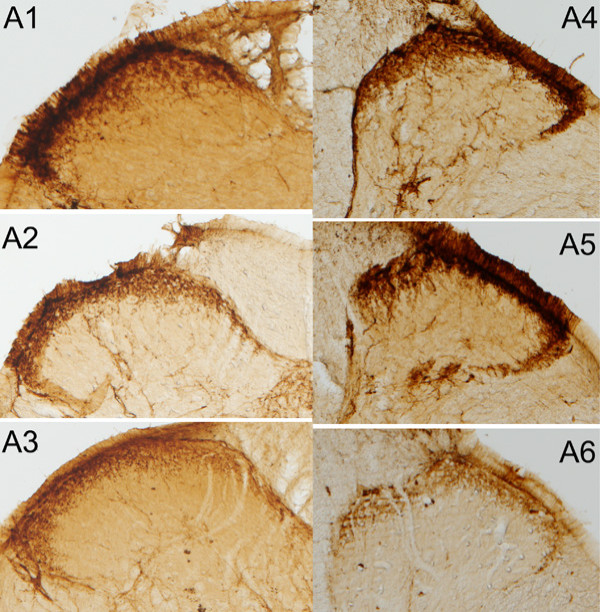
**Photomicrographs of superficial dorsal horn sections**. Examples of photomicrographs of superficial dorsal horn sections immunoreacted for CGRP (A) on days 21 (A_1–3_) and 28 (A_4–6_) for CONT (A_1,4_), DEX (A_2,5_) and CORT (A_3,6_).

**Figure 6:**
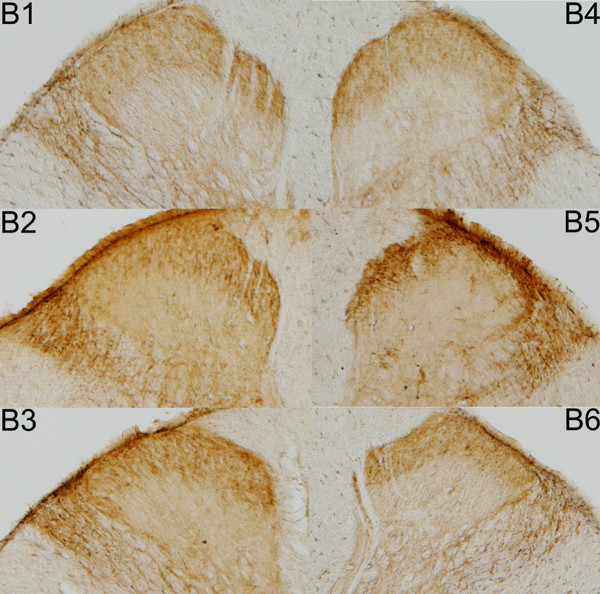
**Photomicrographs of superficial dorsal horn sections**. Examples of photomicrographs of superficial dorsal horn sections immunoreacted for GABA_B2 _(B) on days 21 (B_1–3_) and 28 (B_4–6_) for CONT (B_1,4_), DEX (B_2,5_) and CORT (B_3,6_).

At the end of the experimental period (21 or 28 days), animals were anesthetized intraperitoneally (sodium pentobarbital, 0.5 mg/kg) and perfused transcardially with 4% paraformaldehyde in PBS 0.1 M, pH 7.2. The spinal cord was removed and placed in 30% sucrose for 24 h. Portions of the cervical and lumbar spinal cord enlargements were sampled. Sections, 30 μm thick, were cut on a vibrating blade microtome (Leica, Germany) and collected in superfrosted slides. Sections from the same region for all subjects and treatments were exposed to the same solutions. Sections were permeabilized for 10 min in 0.2% Triton X-100 in Tris buffer saline (TBS) and microwaved (20 min) while immersed in citrate buffer (0.1 M). Endogenous peroxidase activity was blocked with 3% H_2_O_2 _in PBS (10 min) and non-specific staining was blocked with 4% bovine serum albumin (BSA) in PBS (30 min). Alternating sections were incubated overnight at room temperature in rabbit primary antibodies against CGRP (1:3000; Chemicon, USA) (Fig. 5), SP (1:3000; Chemicon, USA), SS (1:3000; Chemicon, USA) and GABA_B2 _(1:1000, Chemicon, USA) (Fig. 6) in 0.02% Triton X-100 (PBST). Antigen visualization was carried out using a universal detection system (BioGenex, San Ramon, CA) and diaminobenzidine (DAB; 0.025% and 0.5% H_2_O_2 _in Tris-HCl 0.05 M, pH 7.2).

### Stereology

The stereological analysis was performed in the dorsal horn of CGRP-, SP-, SS-and GABA_B2_-immunoreacted (IR) spinal cord sections using StereoInvestigator software (MicroBrightField, Williston/VT, USA). From each set of serial sections, ten photomicrographs of areas within the spinal laminae I–II were obtained at a primary magnification of × 50 and analyzed at a final magnification of × 1000. The number of stained fibres per unit of laminae I–II volume (numerical density) was estimated using the optical fractionator method [65]. The surface volume occupied by laminae I–II stained fibres was calculated on the basis of the surface density of the fibres (surface area per unit volume, SV) and the volume of laminae I–II. The SV was estimated, using a 'staggered' cycloid test system in order to obtain the total number of intersections between cycloid arcs and stained fibres. Measurements were made on laminae I–II regions randomly selected by the software.

### Densitometric Measurements

The densitometric analysis was performed in the dorsal horn of CGRP, SP, SS and GABA_B2_-IR spinal cord sections using a Zeiss light microscope coupled to a PC, using NIH Image 1.52 software. The sampling area for optical density measurement corresponded to all the area occupied by laminas I and II of the spinal dorsal horn, bilaterally. Density levels and distribution of CGRP-, SP-, SS- and GABA_B2_-IR were quantified and, for all sections, background density measurements were subtracted to these values.

### Data analysis

Statistical analysis was performed using GraphPad Prism version 4.00 for Windows (GraphPad Software, San Diego California, USA). A two-way ANOVA (ANOVA_2 w_) was used to analyze differences between groups at different time points, while repeated-measures ANOVA (ANOVA_rm_) was used to evaluate efficiency of treatment along different time points within groups; pos-hoc Bonferroni's test was used to detect significant differences for both ANOVA analysis. Densitometric and stereological data was compared using the Pearson correlation analysis. Differences were considered statistically significant when p < 0.05. All values are presented as mean ± SD.

## Abbreviations

CGRP: Calcitonin gene-related peptide; CONT: Controls; CORT: Corticosterone; DEX: Dexamethasone; GABA_B2_: B2-γ-aminobutiric acid receptors; GC: Glucocorticoid; GR: Glucocorticoid receptor; HP: Hot-plate test; IR: Immunoreactivity; MR: Mineralocorticoid receptor; SP: Substance P; SS: Somatostatin; TF: Tail-flick test

## Competing interests

The authors declare that they have no competing interests.

## Authors' contributions

FPR performed the statistics analysis, carried out the IHC and quantification of densitometry and drafted the paper. VM: performed behavioural tests and the stereological analysis. JMP: performed corticosteroid administration and behavioural testing. PL: performed corticosteroid administration and stereological quantification. AA: conceived, designed and coordinated the study and revised the paper. NS: conceived, designed and coordinated the study and revised the paper. All authors read and approved the final manuscript.
